# An Overview of Epigenetics in Obesity: The Role of Lifestyle and Therapeutic Interventions

**DOI:** 10.3390/ijms23031341

**Published:** 2022-01-25

**Authors:** Abeer M. Mahmoud

**Affiliations:** Division of Endocrinology, Diabetes, and Metabolism, Department of Medicine, College of Medicine, University of Illinois at Chicago, Chicago, IL 60612, USA; amahmo4@uic.edu

**Keywords:** obesity, epigenetics, energy metabolism, lifestyle, DNA methylation, histone modifications, microRNAs, diet, physical activity, weight loss

## Abstract

Obesity has become a global epidemic that has a negative impact on population health and the economy of nations. Genetic predispositions have been demonstrated to have a substantial role in the unbalanced energy metabolism seen in obesity. However, these genetic variations cannot entirely explain the massive growth in obesity over the last few decades. Accumulating evidence suggests that modern lifestyle characteristics such as the intake of energy-dense foods, adopting sedentary behavior, or exposure to environmental factors such as industrial endocrine disruptors all contribute to the rising obesity epidemic. Recent advances in the study of DNA and its alterations have considerably increased our understanding of the function of epigenetics in regulating energy metabolism and expenditure in obesity and metabolic diseases. These epigenetic modifications influence how DNA is transcribed without altering its sequence. They are dynamic, reflecting the interplay between the body and its surroundings. Notably, these epigenetic changes are reversible, making them appealing targets for therapeutic and corrective interventions. In this review, I discuss how these epigenetic modifications contribute to the disordered energy metabolism in obesity and to what degree lifestyle and weight reduction strategies and pharmacological drugs can restore energy balance by restoring normal epigenetic profiles.

## 1. Introduction

Over the last century, science has contributed to an almost three-decade increase in the average person’s lifespan. However, the rising obesity prevalence is considered an epidemic that threatens to reduce current and future generations’ life expectancy and quality of life [1]. This will also pose a considerable challenge to future healthcare finances. Obesity increases the risk of various comorbidities, including diabetes, cardiovascular disease, and cancer [2]. Furthermore, recent research has found that obese people are more likely to suffer significant repercussions from the overlapping COVID19 pandemic [3]. Therefore, it is crucial to understand the underlying cellular and molecular mechanisms and identify novel therapeutic targets for obesity.

Genome-wide association studies (GWAS) have revealed several hundred genetic loci that are associated with body mass index (BMI); however, these correlations can only explain around 3–5% of the BMI variance observed in the population [4]. According to the World Health Organization’s (WHO) most recent report, worldwide obesity has nearly tripled since 1975. Overweight people account for more than 1.9 billion (39%) of the adult population, with over 650 million (13%) obese. This enormous increase in obesity rates over the last few decades cannot be explained entirely by genetics. Therefore, scientists should look beyond genetics and consider the influence of lifestyle factors and obesogenic environmental exposures and their potential interaction with the genome.

Obesity is currently considered a pandemic due to the surrounding obesogenic environment, including the availability of calorie-dense fast food and recent technological advances that significantly diminish daily physical activity [5]. Nevertheless, obesity is still avoidable by improved lifestyle and everyday decisions regarding nutrition, physical exercise, and other lifestyle variables. These variables have been found to alter DNA transcription and, consequently, how genes are expressed. This process is referred to as epigenetics, which includes dynamic and adjustable molecular changes that reflect the body’s interaction with its environment [6]. As a first step in addressing the obesity problem, we must understand the impact of lifestyle changes and therapeutic interventions on the epigenetic control of energy expenditure in obesity. In the current review, I will provide an overview of the intricate interplay between epigenetics and energy metabolism in the context of obesity development and management.

## 2. The Role of Epigenetics in Obesity

The significantly increased rate of obesity over the last few decades cannot be totally explained by genetics. Environmental variables such as nutrition and lifestyle may contribute to this tendency. These variables have the ability to change gene expression without affecting the DNA sequence, which is a phenomenon known as epigenetics. Conrad Waddington first introduced the concept of epigenetics in 1942, and it simply refers to chemical modifications that change how the body reads DNA. The most common epigenetic changes that have been thoroughly studied are DNA methylation, histone modifications, and non-coding RNAs [7].

Epigenetics has recently begun to provide explanations for alterations observed in many physiological and pathological processes, and the role of epigenetic modifications in obesity is becoming more apparent. One of the most common examples of epigenetics’ influence on obesity is the agouti mouse model. This model has a mutation in the agouti viable yellow (*Avy*) gene, which controls mouse coat color and is usually imprinted via DNA methylation. Aberrant hypomethylation of this gene causes ectopic expression of its protein, which binds to the melanocortin 4 receptor (MC4R) in the hypothalamus, disrupting its function and triggering hyperphagic obesity. Interestingly, this phenotype was attenuated in the offspring of mice fed a diet rich in methyl donors, indicating the reversibility of epigenetic alterations in response to external factors such as diet. Methyl donors are dietary components such as folate and methionine that provide methyl groups for biological DNA methylation. The diet-induced restoration of DNA methylation improved body weight and insulin sensitivity in the agouti mouse model, establishing the relationship between obesity and environmental-induced epigenetic changes [8].

Several genes implicated in obesity and other metabolic diseases are epigenetically regulated. This section will review current research, highlighting the contribution of major epigenetic alterations, mainly DNA methylation, histone modifications, and non-coding RNAs, to obesity.

### 2.1. DNA Methylation

DNA methylation is an essential epigenetic mechanism, and its role in the pathogenesis of several diseases has been extensively studied. The process of DNA methylation consists of covalent binding of a methyl group to a cytosine residue in the DNA, where cytosines are followed by guanines (CpG sites). This chemical modification is mediated via a group of enzymes called DNA methyltransferases (DNMTs). DNA methylation, especially in the gene promoter regions, interferes with gene transcription. This effect is achieved by preventing transcription factors from accessing the DNA and recruiting transcription-repressive proteins such as histone deacetylases (HDACs). Aberrant DNA methylation could occur at the global or specific gene level. For example, global DNA hypomethylation has been identified as a hallmark of several malignancies; however, a similar relationship in obesity is inconsistent among studies. Some studies observed global DNA hypomethylation in blood and adipose tissue samples from obese individuals [9], while others reported global hypermethylation [10].

Candidate gene methylation, on the other hand, exhibited more consistent findings. Leptin and adiponectin are at the top of the genes linked to obesity. These hormones are predominantly produced by the adipose tissue and regulate energy balance and metabolism. A recent study by Sadashiv et al. [11] reported a negative association between body weight and DNA methylation of the leptin (*LEP*) gene promoter measured in blood in a cohort of obese adults. *LEP* promoter hypomethylation has also been linked to impaired glucose metabolism, decreased insulin sensitivity, and an altered lipid profile [11]. Similarly, in a cross-sectional study by Houde et al. [12], the body mass index of pre-bariatric obese adults correlated negatively with *LEP* methylation and positively with adiponectin gene (*ADIPOQ*) methylation measured in subcutaneous adipose tissues. Several other investigations have confirmed the association between obesity and the methylation status of *LEP* and *ADIPOQ*, which has been proposed to promote obesity-associated insulin resistance and metabolic derangements [13,14,15].

DNA methylation also regulates genes involved in insulin signaling, such as insulin (*INS*) [16], insulin receptor substrate 1 (*IRS1*) [17], and phosphatidylinositol 3-kinase regulatory subunit (*PIK3R1*) [18]. The methylation status of these genes was found to be altered under conditions of obesity and metabolic disease. For example, in a study by Rohde et al. [19], higher *IRS1* promoter methylation and lower gene expression were observed in visceral and subcutaneous adipose tissues of a cohort of 146 obese individuals. In this study, *IRS1* promoter methylation was directly associated with body weight, waist circumference, and indices of impaired glucose metabolism. It was also shown that DNA methylation of *PIK3R1*, an essential gene for insulin signaling, was reduced and its expression was enhanced after weight loss surgery [18]. A strong link was found between these methylation profiles and physiological outcomes such as insulin sensitivity and energy metabolism [17,20].

Several other genes involved in the pathogenesis of obesity are regulated by DNA methylation. Of these are *PGC1A* (peroxisome proliferator-activated receptor γ coactivator 1 alpha), a critical transcriptional factor for energy expenditure, and *IGF-2* (insulin-like growth factor 2), which mediates vital cellular processes such as growth, differentiation, and metabolism. The methylation of these two genes was disrupted in obesity, gestational diabetes, and high-fat feeding but was restored in response to caloric restriction [21,22,23]. *POMC* (Pro-opiomelanocortin) and *NPY* (Neuropeptide Y) are two appetite-regulating genes whose methylation is altered in obesity; *POMC* promotes satiety, while *NPY* stimulates food intake. Weight gainers and individuals resistant to weight loss interventions had higher *POMC* methylation levels and lower *NPY* methylation levels [24].

Genes involved in hypoxia and inflammation are another example of altered DNA methylation in obesity. On top of these genes is *HIF3A* (hypoxia-inducible factor 3a), which is an essential gene in metabolic and physiological responses to hypoxia whose methylation has been linked to obesity in both adults and children [25,26]. In addition, genes involved in inflammation and oxidative stress such as *TNF* (tumor necrosis factor), *IL6* (interleukin 6), and *TFAM* (mitochondrial transcription factor A) exhibited aberrant DNA methylation in obese individuals and correlated with abnormal expression and disturbed function of these genes [27,28,29]. Collectively, obesity has been linked to altered DNA methylation patterns, which could serve as biomarkers and therapeutic targets. These changes have been linked to a disrupted balance between DNA methyltransferases (DNMTs) and active demethylases such as TETs (Ten-eleven translocation methylcytosine dioxygenase 1) [28,29,30,31]. However, further research is needed to identify the underlying mechanisms of aberrant DNA methylation in obesity.

### 2.2. Histone Modifications

Histones are globular proteins around which DNA is wrapped to produce compact chromatin. There are five families of histones known as H1, H2A, H2B, H3, and H4. Post-translational modifications of these histones, including acetylation, methylation, phosphorylation, and ubiquitination, influence the compactness of DNA packing and hence the accessibility of transcription factors and subsequent gene expression. For example, histone acetylation stimulates gene expression by relaxing the DNA–histone interaction, rendering a permissible chromatin structure, whereas histone methylation can either activate or repress DNA transcription depending on the location and number of methylated residues [32]. Histone deacetylases (HDACs), histone acetyltransferases (HATs), histone demethylases (HDMs), and histone methyltransferases (HMTs) are among the enzymes responsible for histone modifications. Changes in these enzymes have been observed in obesity and linked to the altered expression of several genes involved in adiposity and metabolic functions [33]. HDACs and the histone 3 lysine 9 (H3K9)-specific demethylase, Jhdm2a, are at the top of the list of epigenetic modifiers that have been shown in clinical and mechanistic studies to accelerate the progression of obesity [34,35].

Histone modifications were shown to regulate the expression of critical genes in adipogenesis such as CCAAT enhancer-binding protein β (*C*/*EBPB*), *C*/*EBPA*, preadipocyte factor- 1 (*Pref-1*), adipocyte protein 2 (*aP2*), and peroxisome proliferator-activated receptor γ (*PPARG*) [36]. In addition to the DNA methylation discussed above, histone acetylation regulates the gene expression of the appetite-regulating genes *POMC* and *NPY*. Reduced acetylation of H3K9 at the *PMOC* and increased acetylation of the same residue at the *NPY* gene have been associated with high-fat diet-induced obesity [37]. Increased acetylation of H3K9 and H3K18 at the *TNF* (tumor necrosis factor) and *CCL2* (monocyte chemotactic protein 1) genes was also seen in the livers of high-fat diet-fed animals and is likely to be responsible for the induced inflammation [37]. On the other hand, caloric restriction and weight loss interventions reversed this pattern and elevated H4 acetylation and thus the expression of glucose transporter 4 (*GLUT4*) in adipose tissues [38]. These findings imply that histone alterations are involved in the epigenetic regulation of adipogenesis and may play a role in the development and progression of obesity.

### 2.3. Non-Coding RNAs

Non-coding RNAs are RNA molecules that are not translated into proteins but have a regulatory impact on gene expression. The most functionally relevant types of non-coding RNAs studied in the context of obesity are microRNAs (miRNAs) and long non-coding RNAs (lncRNAs). MicroRNAs are small molecules of RNA (≈22 nucleotides) that exert their function via post-transcriptional regulation of gene expression. Several miRNAs are involved in the process of adipogenesis, such as *miR-30*, *miR-26b*, *miR-199a*, and *miR-148a*. These miRNAs were found in higher levels in obese individuals and mice fed a high-fat diet. Similarly, *miR-17-5p* and *miR-132* were expressed at higher levels in the visceral adipose tissues of obese adults and correlated significantly with body mass index, glycosylated hemoglobin, and impaired glucose and lipid metabolism [39].

An induced expression of *miR21* was found in the white adipose tissues of obese people compared to lean controls and correlated with impaired vascular function. Leptin-targeting *miR221* was upregulated in obese people and diet-induced obese mice. In mechanistic models where these miRNAs were functionally silenced, reductions in the process of adipogenesis and triglyceride accumulation were observed, as well as improvements in body weight and metabolic function. A large number of miRNAs involved in adipogenesis, fat metabolism, insulin signaling, hypoxia, inflammation, and cell development and differentiation were discovered to be differently expressed in obese people. Landrier et al. [39] provide a comprehensive list of miRNAs related to obesity and metabolic diseases.

More research has recently begun to unravel the biological functions of lncRNAs, which are tissue-specific long RNA transcripts (>200 bp) that do not encode proteins but regulate gene expression. *GYG2P1*, *lncRNAp21015*, and *lncRNA-p5549* are examples of lncRNAs that are differentially expressed in obesity [40,41]. The expression level of these lncRNAs was reduced in obese individuals and correlated adversely with body mass index, waist circumference, fasting insulin, and triglycerides. *RP11-20G13.3* is among the lncRNAs required to maintain *PPAR*, *C/EBP*, and *ADIPOQ* levels during adipogenesis and is differentially expressed in obesity [40]. Other lncRNAs, such as *lnc-dPrm16* and *MIST*, have been shown to influence brown adipogenesis, inflammation, and lipid metabolism [42,43]. Squillaro et al. [44] reported several other lncRNAs that contribute to obesity in a comprehensive review article, adding to the mounting evidence for the role of non-coding RNAs in obesity.

## 3. Epigenetic Effects of Environmental Factors and Lifestyle

Epigenetic mechanisms are dynamic and modifiable in response to environmental factors and lifestyle, allowing for adaptation to external stimuli and the restoration of standard epigenetic profiles when these stimuli are removed. The current section will discuss examples of environmental exposures, lifestyle factors, and therapeutic interventions that were shown to modify epigenetic mechanisms implicated in obesity.

### 3.1. Obesogenic Exposures

In 2002, Baillie-Hamilton et al. [45] brought to the scientific community’s attention the fact that the start of the obesity epidemic coincided with the beginning of the industrial revolution and the use of several endocrine disruptors such as organophosphate pesticides, bisphenol A, solvents, chemicals, and heavy metals. Later on, Grun and Blumberg [46] developed the term “environmental obesogens,” referring to environmental exposure to endocrine disruptors that induce adiposity via disrupting the body’s natural mechanisms of weight control.

Bisphenol A (BPA), a synthetic compound present in beverage containers, water bottles, and dental materials, is one of the most widespread endocrine disruptors. Individuals with high plasma levels of BPA were more likely to develop visceral obesity, insulin resistance, and metabolic disorders [47]. Choi et al. [48] reported higher body mass index and differential methylation in the insulin growth factor 2 receptor (*IGF2R*) gene in children exposed to high BPA prenatally. Mechanistic studies in Agouti mice exposed to BPA revealed promoter hypomethylation and enhanced expression of the *Agouti* gene [8]. These mice were predisposed to obesity, diabetes, dyslipidemia, and other metabolic abnormalities. Furthermore, transient exposure to low doses of BPA altered global methylation, *PPARG* promoter methylation, and histone methylation (H3K4, H3K9, and H3K36) in cultured preadipocytes and other in vitro models [49,50,51].

Phthalates are chemical compounds used in plastic products such as medical devices and children’s toys; they have endocrine-disrupting effects. A significant correlation was reported between urinary levels of phthalates and metabolic disorders, including obesity and insulin resistance. At the epigenetic level, phthalates were found to alter the methylation of metabolic genes such as *PPARG*, insulin growth factor 2 (*IGF2*), and sterol regulatory element-binding proteins (*SREBPs*) [52]. Furthermore, phthalates were found to stimulate the expression of *miR-34a-5p* while decreasing the expression of its target genes, nicotinamide phosphoribosyltransferase (*NAMPT*) and sirtuin 1 (*Sirt1*), both of which are essential for energy homeostasis [53]. The *lncRNA H19* and its downstream pathway related to insulin signaling were also altered in response to phthalates [54].

Organochlorine and organophosphate pesticides were found to accumulate in the adipose tissues and adversely affect metabolic pathways such as PPARγ and hepatic adenylyl cyclase/cyclic AMP signaling and inflammatory cytokines via mechanisms that involve global hypomethylation and aberrant histone methylation (H3K27) [55]. Growing epidemiological evidence supports the contribution of these pesticides to the global rise in obesity and diabetes [56]. Several other toxic environmental particles, industrial inhaled pollutants, and flame retardants were found to alter the DNA methylation status of *PPARG* and downstream pathways contributing to systemic inflammation and insulin resistance observed in obesity, diabetes, and other metabolic disorders.

### 3.2. Dietary Factors

Obesity is not simply the outcome of excess food intake or energy imbalance. Instead, numerous epigenetic mechanisms are implicated in diet-induced obesity. Association studies have linked specific dietary patterns with DNA methylation profiles in humans, as reported by Maugeri and Barchitta [57]. For example, a study by Piyathilake et al. [58] observed decreased global DNA methylation, as measured by the methylation level of long interspersed nucleotide element-1 (*LINE-1*) in the blood of women who consume high-fat meals versus those who follow healthy dietary patterns. A few clinical trials have further supported the link between high-fat diet consumption and DNA methylation. Brøns et al. [22] found that a five-day high-fat diet feeding increased DNA methylation of the transcription factor *PPARG*, which influences mitochondrial activity and energy metabolism. This impact was more pronounced in people with normal birth weight than those with low birth weight, who had higher *PPARG* methylation levels at baseline. The effect of a short-term high-fat diet was also observed in a genome-wide methylation analysis by Jacobsen et al. [59], who found alterations in DNA methylation in 6,508 genes implicated in inflammation, cancer, and reproduction in skeletal muscles of healthy young men.

Preclinical studies demonstrated that hypercaloric and high-fat diets modulate the methylation of genes involved in metabolism and appetite regulation, such as leptin (*LEP*) in the adipose tissues and melanocortin 4 receptor (*MC4R*) in the brain [60]. Furthermore, elevated expression of the histone deacetylases HDAC5 and HDAC8 was observed in the ventromedial hypothalamus of rodents fed a high-fat diet, which subsequently dysregulated a myriad of genes responsible for energy metabolism and appetite regulation [34]. Chronic high-fat diet intake was also shown to induce HDAC9 that interfered with adipocyte differentiation, resulting in improperly differentiated adipocytes low in adiponectin and inefficient at storing lipids [61]. The epigenetic impact of a high-fat diet has been demonstrated to be mediated, at least partly, by HDAC11, which is a thermogenesis repressor gene that downregulates thermogenin/*UCP1*, *PGC1A*, and *BRD2* (Bromodomain-containing protein 2). HDAC11-deficient animals showed reduced body weight and fat mass and increased thermogenic capacity, metabolic rate, and physical activity when fed a high-fat diet compared to their wild-type counterparts [62].

The effects of a high-fat diet have been found to extend into the prenatal period. A high-fat maternal diet in rodents was shown to influence the hypothalamic regulation of energy metabolism and body weight in the offspring by causing epigenetic changes in the *POMC*, *NPY*, and *LEPR* genes. These changes were associated with the prevalence of obesity, dyslipidemia, steatohepatitis, insulin resistance, hyperglycemia, hyperleptinemia, and inflammation in the offspring, which lasted into adulthood [63]. A maternal high-fat diet for four weeks in mice caused hypermethylation of the *IRS2* (insulin receptor substrate 2) gene and hypomethylation of the *MAP2K4* (mitogen-activated protein kinase 4) gene in the offspring. These modifications were associated with significant changes in *IRS2* and *MAP2K* mRNA levels, as well as increased body weight, hyperglycemia, and insulin resistance [64]. The favorable epigenetic consequences of the prudent diet are supported by accumulating epidemiological evidence. For example, a cross-sectional study in Southern Italy by Zhang et al. found that consuming a balanced nutritious diet rich in fruits and vegetables was strongly associated with global DNA methylation in peripheral blood. Similar results were reported in the North Texas Healthy Heart Study [65].

Carbohydrates are a primary macronutrient in our diet that supply energy and hence can contribute to excessive energy consumption and weight gain. However, carbohydrates have a lower energy density than fat. When compared to low-fat diet trials, low-carbohydrate diet studies showed mixed results in terms of weight loss and other health benefits. The reason behind this inconsistency is that our diets contain a wide variety of carbohydrates that have different glycemic indices and variable effects on our health. While sugar-sweetened beverage consumption has been associated to weight gain, dietary fibers and sugars from whole grains, fruits, and vegetables are significant sources of energy and are required for various physiological functions, including the microbiome [66].

Few studies have explored the influence of carbohydrate intake on inducing epigenetic changes that might alter the risk of obesity. For example, Lai et al. [67] reported a link between carbohydrate intake as well as the carbohydrate/fat ratio and a higher methylation status of *CPT1* (Carnitine palmitoyltransferase-1) gene. *CPT1* is involved in appetite regulation and insulin-mediated glucose and fatty acid metabolism, and its DNA methylation levels have been associated with a decreased risk of obesity. Similar results were seen in animal models fed a high-carbohydrate, low-fat diet, which increased *CPT1* methylation in liver tissue and protected them from weight gain [68,69]. A study by Ramos-Lopez et al. [70] discovered a link between carbohydrate intake and the methylation state of the *DA* (dopamine) gene, which codes for a neurotransmitter that regulates the central reward system and food intake. Hypermethylation and *DA* downregulation have previously been connected to eating disorders and obesity indicators. Furthermore, DNA methylation of circadian clock genes such as *RORA* (RAR Related Orphan Receptor A) and *BMAL1* (brain and muscle aryl hydrocarbon receptor nuclear translocator–like 1), which control body metabolism, was found to be associated with carbohydrate consumption but not with protein or fat-rich diets. [71,72]. However, studies comparing the epigenetic impacts of various carbohydrate types are lacking. Furthermore, epigenetic alterations as a potential mechanism for the obesogenic effects of currently used sugar additives and artificial sweeteners need to be investigated.

Inadequate early nutrition, as seen in hypocaloric and low-protein diets, has also been linked to obesity, diabetes, hypertension, and hypercholesterolemia [73]. The Dutch famine in the winters of 1944 and 1945 is the best demonstration of this phenomenon [74]. These effects were reproduced in animal models, which aided our understanding of the epigenetic alterations responsible for the observed phenotypes. In these models, the offspring of mothers fed a hypocaloric, low-protein diet exhibited altered methylation of the hepatic *PPARG* and adrenal *IGF2* genes and induced H4 acetylation of the *GLUT4* and *C*/*EBPB* genes. These changes appear to rewire orexigenic pathways and the brain’s reward center, leading to binge eating, obesity, and metabolic abnormalities in the offspring [75,76].

On the other hand, some micronutrients boost body metabolism and energy homeostasis by inducing favorable epigenetic alterations. Short-chain fatty acids (e.g., butyrate) and polyunsaturated fatty acids are examples of these nutrients, as they exert anti-inflammatory effects via modifying HDAC activity and DNA methylation [77]. During the one-carbon metabolism cycle, dietary methyl donors such as folate, methionine, choline, and betaine transfer a methyl group to the DNA and histones, contributing to epigenetic modifications and gene expression changes. Vitamins including B_2_, B_6_, and B_12_ and minerals such as zinc and selenium are cofactors in the one-carbon cycle and contribute to epigenetic changes as well [78]. A cross-sectional study by Ramos-Lopez et al. [79] described an association between folate deficiency in obese subjects and hypomethylation of the *CAMKK2* (calcium/calmodulin-dependent protein kinase 2) gene that regulates energy metabolism and insulin sensitivity. The insulin resistance index, HOMA-IR, correlated negatively with *CAMKK2* methylation and positively with its mRNA expression.

However, the direct correlation between folate and methyl donor consumption and global DNA methylation has not always been constant across investigations; some have observed inverse [80] or even null associations [20,81]. This discrepancy might be attributed to differences in participant characteristics across studies or inaccuracies in reporting food consumption. Some dietary nutrients, such as calcium, vitamin C, and vitamin E, have been demonstrated to promote epigenetic modifications that aid in weight regulation. In contrast, magnesium and chromium have been shown to induce alterations that predispose to obesity [82]. As a result, several of these micronutrients may be effective as epigenetic therapies. Nevertheless, further research is needed to understand their underlying mechanisms and the optimal doses considered to be protective.

The term “epigenetic diet” refers to bioactive nutritional compounds that can induce epigenetic changes and alter gene expression. This ever-expanding list of epigenetic nutrients includes minerals, vitamins, polyphenols, and various phytochemicals. Among these phytochemicals are resveratrol (grapes), tea catechins and polyphenols (green tea), genistein and quercetin (soybean), curcumin (turmeric), sulforaphane (cruciferous vegetables), and diallyl disulfide (garlic). However, most of the research to date has focused on the effect of these nutrients on cancer progression, and additional research is needed to determine a comparable protective role in obesity and metabolic diseases. Herein, I discuss a few examples of these phytochemicals reported to modulate body weight and energy expenditure via epigenetic mechanisms.

The tea catechin epigallocatechin-3-gallate (EGCG) has been shown to reduce body weight, blood glucose and insulin levels, liver triglycerides, cholesterol, and circulating inflammatory cytokines in rodent models. It was proposed that EGCG’s ability to induce these effects was mediated by suppressing DNMT and histone acetyltransferase activity [83]. Another example of epigenetic micronutrients is dietary isoflavones. When administered at low doses, the soy isoflavone genistein enhanced body weight, lipid profile, and insulin sensitivity. This effect was mediated mainly by changes in the DNA methylation of multiple genes in the liver and muscles [82]. Curcumin, a turmeric polyphenol, has been shown in numerous studies to inhibit adipogenesis by increasing fatty acid oxidation and suppressing lipogenic and inflammatory gene expression. These effects were demonstrated to be mediated by changes in the activity of DNMTs, histone deacetylases and acetyltransferases, and many miRNAs [84].

Resveratrol, a well-known free radical scavenger that significantly improves cardiovascular and metabolic functioning, is a potent activator of the (NAD(+))-dependent histone deacetylase, sirtuin 1. Resveratrol has been proven in numerous studies to improve hepatic steatosis, insulin sensitivity, and raise the lipolysis/lipogenesis ratio, hence improving body weight in a manner comparable to caloric restriction diets [85]. Another example is the organosulfur compounds such as sulforaphane (cruciferous vegetables) and diallyl disulfide (garlic), which suppress adipogenesis and inflammation via modifying histone acetylation [86]. Overall, despite significant evidence for the anti-obesity benefits of these dietary components in animal models, there is a lack of intervention trials investigating their effects in humans.

### 3.3. Physical Activity

A sedentary lifestyle contributes to obesity via reducing insulin sensitivity, energy metabolism, mitochondrial function, and redox homeostasis. A growing body of research suggests that exercise can reverse these morbidities by triggering epigenetic modifications. Bajpeyi et al. [87] observed reductions in DNA methylation in the regulatory region of the *PGC1α* gene. This transcriptional factor regulates energy expenditure and mitochondrial biogenesis in skeletal muscle biopsies from exercising subjects. These findings were linked to enhanced *PGC1α* gene expression and lower intramyocellular lipid levels.

Genome-wide methylation analysis of adipose tissues after six months of endurance training demonstrated changes in the DNA methylation of 63 genes involved in obesity and diabetes, including *KCNQ1* (potassium voltage-gated channel subfamily Q member 1), *HHEX* (haematopoietically expressed homeobox), *IGF2BP2* (insulin-like growth factor 2 mRNA binding protein 2), *JAZF1* (JAZF zinc finger 1), and *TCF7L2* (transcription factor 7 like 2) [88]. Rowlands et al. [89] analyzed DNA methylation and miRNA expression in skeletal muscle biopsies obtained from sedentary obese and diabetic subjects before after 16 weeks of either aerobic or resistance training. Aerobic exercise decreased DNA methylation of *NRF1* (nuclear respiratory factor 1), a transcription factor for essential metabolic genes, and increased the methylation of the *FASN* (fatty acid synthase) gene, resulting in improvements in metabolic functions and reductions in circulating lipids. The same intervention modified *miR-29a* and *miR-132* that regulate genes involved in lipid and glucose metabolism and vascular function. Resistance training, on the other hand, modified metabolism-related miRNAs (i.e., *miR-1207-5p* and *miR-195*) and increased the methylation of *SLC2A4* (solute carrier family 2), which is a gene that is important in fatty acid metabolism, mitochondrial function, and oxidative capacity. These modifications were associated with better glucose utilization and lower intramuscular lipids [89].

Several modes and intensities of exercise training have been demonstrated to be effective in restoring epigenetic patterns in humans. For example, 60 min of acute endurance exercise increased the expression of *miR-1* and *miR-133a*; both are critical for mitochondrial biogenesis [90]. Similarly, a single bout of acute exercise (10 min in total; 70–90% of maximum heart rate) increased several members of the *miR-378* family known to regulate the *PGC1B* gene, resulting in improvements in mitochondrial function, fatty acid oxidation, and insulin sensitivity [91]. Acute exercise training (80% of maximum oxygen consumption (VO_2max_)) was also shown to decrease DNA methylation at the promotors of *PGC1A*, *TFAM* (transcription factor A, mitochondrial), *PPARD* (peroxisome proliferator-activated receptor delta), *PDK4* (pyruvate dehydrogenase kinase 4), and *CS* (citrate synthase) genes. These genes play essential roles in mitochondrial activity, insulin sensitivity, and energy expenditure [92].

Short-term rehabilitation training (4 weeks) was found to alter the methylation of the *AMPKA2 (PRKAA2;* protein kinase AMP-activated catalytic subunit alpha 2) gene, which is a very important energy sensor that regulates fatty acid metabolism and insulin sensitivity [93]. In addition, short-term training (10 days at 75% VO_2max_) effectively increased the expression of *miR-133a*, *miR-133b*, and *miR-1* and reduced the level of *miR-9*, *miR-23a*, *miR-23b*, and *miR-31*. These changes enhanced mitochondrial biogenesis, muscle growth, and metabolic adaptations in response to exercise training [94]. On the other hand, long-term endurance training (6 months) was shown to restore the DNA methylation profiles of genes involved in calcium signaling, glucose transporter 4 (GLUT4) translocation, and retinol metabolism in skeletal muscles [95]. In diabetic patients, 16 weeks of endurance training-induced DNA hypomethylation in the *PFKFB3* (6-phosphofructo-2-kinase/fructose-2,6-biphosphatase 3), *HDAC4* (histone deacetylase 4), and *GSK3A* (glycogen synthase kinase 3 alpha) genes responsible for glycolysis, transcriptional regulation, and glycogen synthesis, respectively [89]. According to a genome-wide methylation analysis, resistance training at 80% of the one-repetition maximum changed 57,384 CpG sites in peripheral white blood cells. These locations have been assigned to genes involved in metabolic and calcium signaling pathways [96]. Several other studies summarized by Barrón-Cabrera et al. [97] have found that exercise training considerably promotes favorable epigenetic profiles that lower systemic inflammation and vascular remodeling in humans.

### 3.4. Sleep Deprivation

Recently, we began to recognize that factors other than eating more and exercising less may contribute to obesity. Previous research has described the impact of sleep disturbances and night shifting on the development of obesity, type 2 diabetes, and hypertension [98,99,100]. On the other hand, multiple studies have linked sleep deprivation to alterations in the epigenome, notably genes that influence circadian rhythm and are known to affect several metabolic functions [101,102]. Taken together, these findings suggest that sleep disturbances may trigger epigenetic changes that contribute to obesity and metabolic diseases. However, there has been a scarcity of research specifically investigating this triangular relationship.

The circadian rhythm is a translational–transcriptional feedback loop composed of positive transcriptional regulators such as CLOCK (circadian locomotor output cycles kaput) and BMAL1 (brain and muscle aryl hydrocarbon receptor nuclear translocator–like 1) and negative transcriptional regulators such as period 1, 2, and 3 (Per1, Per2, and Per3) and cryptochrome 1 and 2 (Cry1 and Cry2). This molecular clock machinery synchronizes the whole-body metabolism via regulating the expression of many genes, which are called clock-controlled genes. The transcription of these genes has been shown to fluctuate in a circadian manner that is responsive to day/night cycles, diet, physical activity, and other lifestyle and environmental factors.

A single night of sleep deprivation was found to increase DNA methylation in the promoter of the *Cry1* gene and the enhancer region of the *Per1* gene in healthy men’s adipose tissues. These findings were associated with decreased expression of the *BMAL1* and *Cry1* genes in skeletal muscles, as well as elevated blood cortisol levels and impaired glucose tolerance [103]. These observations were in line with prior findings by Zhu et al. [104], who reported *CLOCK* promoter hypomethylation and *Cry2* hypermethylation in long-term night-shift workers. In addition to circadian genes, one-night sleep deprivation was found to induce promoter hypermethylation of *SCD1* (Stearoyl-CoA Desaturase 1), a critical gene in lipid metabolism [105]. Furthermore, multiple studies have shown sleep deprivation to enhance the expression of the DNA methyltransferases DNMT3a1 and DNMT3a2, causing aberrant methylation of thousands of genes [106,107,108].

Changes in DNA methylation profiles in response to sleep deprivation were tissue-specific. For example, Cedernaes et al. [109] reported that acute sleep deprivation resulted in 148 differentially methylated regions (DMRs) in subcutaneous adipose tissue. Still, no significant DMRs were observed in skeletal muscle tissues in this study. The impact of sleep loss on DNA methylation was also gene-specific. Some genes exhibited DNA hypermethylation such as *CD36* (fatty acid transporter), *AKR1CL1* (Aldo-keto reductase family 1 member C8), and *HOXA2* (homeobox A2), *TRIM2* (tripartite motif-containing 2), and *FOXP2* (forkhead box P2) genes involved in lipid metabolism, cell signaling, DNA repair, and adipogenesis. Other genes such as *INS* (insulin), *GFI1* (growth factor independent 1 transcriptional repressor), *CPT1A* (carnitine palmitoyltransferase 1A), *ADORA2A* (adenosine A2a receptor) implicated in insulin signaling, lipolysis, metabolism, and fat beiging showed DNA hypomethylation.

Sleep deprivation has also been demonstrated to increase HDAC1/2 expression, resulting in decreased histone acetylation [110]. Indeed, the circadian rhythm genes *CLOCK*, *BMAL1*, and *Per1* are heavily regulated by rhythmic cycles of histone acetylation and deacetylation, in which HDAC1/2 and SIRT1 (sirtuin 1) play key roles [111]. In addition to disturbances in DNA methylation and histone acetylation, sleep loss induced *lncRNA 116HG* and *A230107N01Rik*, which were found to dysregulate circadian genes *CLOCK*, *Cry1*, and *Per2* [112]. Furthermore, sleep deprivation was found to induce *miR-192*/*194*, *miR-142-3p*, *miR-138*, and *miR-182*, which modulate *ADCY6* (adenylate cyclase 6) and the circadian genes, *CLOCK*, *BMAL1*, and *Per1-3* [113,114,115]. These findings confirm the role of sleep deprivation in the development of obesity, most likely via disrupting the circadian clock genes.

### 3.5. Alcohol Intake

Several studies have linked excessive alcohol use to a higher BMI, explaining this by the fact that each gram of alcohol provides 7.1 Kcal [116]. Aside from providing extra calories, alcohol has various epigenetic effects that may modify the risk of obesity. Ethanol has been found to disrupt the one-carbon metabolism cycle required for DNA methylation by inhibiting the enzyme methionine synthase and reducing the absorption of methyl donors such as folate and other B vitamins [117]. As a result, it is expected that excessive ethanol consumption will interfere with DNA methylation. Indeed, in prior research, my research group found a link between heavy alcohol consumption and DNA hypomethylation in peripheral blood as well as higher homocysteine levels in a cohort of morbidly obese individuals [118].

Acute alcohol intake was demonstrated to reduce global DNA methylation by inhibiting DNMTs. In contrast, chronic alcohol consumption resulted in apparent hypermethylation at global and gene-specific levels [119,120]. Examples of hypermethylated genes in response to chronic alcoholism are *POMC*, which promotes satiety, and alpha-synuclein (*SNCA*) that participates in glucose metabolism and insulin sensitivity in adipose tissues and skeletal muscles [121]. Several other genes involved in metabolism and the central reward center, including *BDNF* (brain-derived neurotrophic factor), *ALDH1L2* (aldehyde dehydrogenase), *GABRP* (GABA receptor), *GAD1* (glutamate-decarboxylase), and *DBH* (dopamine beta-hydroxylase), revealed abnormal DNA methylation in GWAS comparing alcoholics to non-alcoholics [122].

Alcohol has also been demonstrated to modify chromatin via changing histone methylation and acetylation. Chronic alcohol abuse resulted in global and gene-specific histone 3 methylation in the human cortex (H3K4me3) [123]. In animal models, chronic alcohol consumption decreased the methylation/acetylation ratio of the H3K9 residue at the *NR2B* (ionotropic glutamate receptor subunit) gene promoter, causing alterations in energy metabolism [124]. Furthermore, ethanol increased HDAC2 activity in the amygdala and altered histone acetylation at genes involved in appetite regulation, including *CBP* (sarcoplasmic calcium-binding protein) and *NPY*. Alcohol also altered the expression of numerous microRNAs implicated in the activity or reward center, alcohol dependence, and energy expenditure, such as *miR-9*, *miR-7*, and *miR-134* [122].

### 3.6. Weight Loss Interventions

Several epigenetic alterations were discovered in response to weight loss interventions. These changes are promising candidates for use as biomarkers that could distinguish responders from non-responders or help design unique weight loss strategies for each individual. On top of these alterations, promoter methylation of the inflammation-related gene, *TNF* (tumor necrosis factor), has lately gained attention as a possible biomarker of weight loss. Campión et al. [125] observed a dramatic reduction in *TNF* promoter methylation in the peripheral blood of subjects, who lost significant weight following eight weeks of a hypocaloric diet. Similar findings have been described by Cordero et al. [126], in which weight loss responders to a hypocaloric diet had reduced methylation of *TNF* and *LEP* genes in adipose tissues compared to non-responders.

DNA methylation array studies reported several differentially methylated genes among weight loss responders and non-responders. *KCNA3* (potassium voltage-gated channel subfamily A member 3), *INSM1* (insulinoma associated repression factor 1), *NFIX* (nuclear factor I X), *ETS* (V-ets avian erythroblastosis virus E26 oncogene homolog 1), and *GLIS3* (GLIS family zinc finger 3) are examples of genes that were differentially methylated in responders’ adipose tissues [127]. Similarly, the 18-month randomized controlled trial (CENTRAL) of the Mediterranean/low-carbohydrate or low-fat diet revealed substantial differences in DNA methylation between responders, who dropped more than 16% of their body weight, and non-responders. *NUDT3* (nudix hydrolase 3), *NCOR2* (nuclear receptor corepressor 2), *LRRC27* (leucine-rich repeat-containing 27), *CRISP2* (cysteine-rich secretory protein 2), and *SLFN12* (schlafen family member 12) genes, which regulate cell adhesion, metabolism, chromatin modification, and calcium signaling, were among those with significant changes in DNA methylation [128].

Circadian clock genes have recently been found to influence metabolic rhythmicity, and epigenetic changes may play a role in their regulation. Differences in the methylation of critical circadian genes such as *CLOCK* (clock circadian regulator), *BMAL1* (aryl hydrocarbon receptor nuclear translocator-like), and *PER2* (period circadian clock 2) were found in the blood of obese and non-obese people and after a 16-week weight loss program, implying the validity of epigenetic marks as weight loss biomarkers [129]. Moreover, research has revealed that DNA methylation of the appetite-regulation genes, *POMC* and *NPY*, could serve as a biomarker of weight loss maintenance versus weight regain. Lower *POMC* methylation levels in the blood were connected to weight loss maintenance, but lower *NPY* methylation predicted weight rebound 32 weeks after a diet-based weight loss intervention [24]. Several studies have confirmed weight loss programs’ potential to restore epigenetic alterations caused by an obesogenic diet and sedentary behavior. For example, following six weeks of an isocaloric balanced diet, alterations in DNA methylation that affected 6,508 genes in skeletal muscle were restored in humans [59]. Similarly, in animal models of high-fat diet-induced obesity, switching to a balanced chow diet restored standard methylation patterns of *LEP* promoters, *SREBF1* (sterol regulatory element-binding transcription factor 1), *PGC1A*, and *FASN* [130].

Beyond diet and exercise trials, bariatric surgery is another weight loss strategy with long-term benefits such as enhanced insulin sensitivity, metabolism, and cardiovascular function. While changes in global DNA methylation varied between studies, there were consistent alterations in the DNA methylation of specific genes in muscle tissues such as *PGC1α*, *PDK4* (pyruvate dehydrogenase kinase 4), and *SORBS3* (sorbin and SH3 domain containing 3) after bariatric surgery [131,132]. These genes participate in metabolic pathways, cytoskeletal organization, cell adhesion, and cell signaling. DNA methylation changes after bariatric surgery were also identified in liver and adipose tissues for genes involved in insulin signaling and non-alcoholic fatty liver disease pathways [133,134]. In genome-wide analyses, after bariatric surgery, the differentially methylated regions (DMRs) were enriched in metabolic genes such as *SCD* (stearoyl CoA desaturase-1) that converts saturated fatty acids into monounsaturated fatty acids and has also been clinically linked to insulin sensitivity and circulating levels of free fatty acids and adiponectin [135]. These DMRs were also enriched in inflammation-related genes such as *SERPINE-1* (serpin family E member 1), *IL-6* (interleukin 6), *TNF*, *IL-1B*, and *PKD4* [136]. In addition to DNA methylation, bariatric surgery has been shown to induce modification in miRNAs and lncRNAs that regulate the cell cycle, cell development, lipid metabolism, inflammatory response, insulin resistance, and endocrine function [137,138,139]. Overall, these findings suggest that weight loss is associated with the reversal of obesity epigenetic patterns and the acquisition of more favorable profiles associated with improved inflammatory, metabolic, and vascular function.

### 3.7. Epigenetic Drugs

In addition to being biomarkers of obesity progression and response to interventions, the epigenetic modifications discussed above serve as therapeutic targets for epigenetic modifiers. While these medications have long been approved for cancer treatment, their potential effects in restoring energy homeostasis and boosting metabolic function have only lately come to light. These drugs target histone deacetylases (HDACs) and demethylases (HDMs), DNA methyltransferases (DNMTs), and protein arginine methyltransferases (PRMTs). Among these drugs, HDAC inhibitors have received the most attention due to their effects on adipogenesis and insulin sensitivity. Valproic acid, a short-chain branched fatty acid with HDAC inhibitory action, was shown to modify hepatic gluconeogenesis and fatty acid oxidation, lowering blood glucose while improving hepatic lipid metabolism and reducing liver steatosis in obese mice [140], making valproic acid a good candidate for use as an adjuvant treatment in obesity and diabetes.

Sodium phenylbutyrate is another HDAC inhibitor that maintains cellular homeostasis by regulating protein folding, cell signaling, and endoplasmic reticulum stress. Although sodium phenylbutyrate did not reduce body weight in obese mice, it increased insulin and leptin sensitivity and decreased blood glucose, inflammation, and endoplasmic reticulum stress indicators [141]. Vorinostat and givinostat are orally active HDAC inhibitors that are effective in treating cancer but are currently being studied as a potential treatment for obesity and diabetes-related morbidities. Both inhibitors have been shown to reduce inflammation by suppressing inflammatory cytokines and transcriptional factors such as NFκβ (nuclear factor κ B), IFNγ (interferon-gamma), IL-1β (interleukin 1 beta), and TNFα (tumor necrosis factor-alpha) [142,143,144]. Givinostat increased pancreatic cell survival, glucose disposal, and insulin sensitivity and lowered inflammatory cytokines in a streptozotocin-induced diabetes mouse model, indicating its potential application in type 1 and type 2 diabetes [145].

Of the above-mentioned HDAC inhibitors, valproic acid and sodium phenylbutyrate have been tested in clinical trials for obesity and diabetes. In a clinical trial by Xiao et al. (NCT00533559; https://clinicaltrials.gov/; accessed on 14 January 2022) [146], pretreatment with sodium phenylbutyrate protected subjects from β cell dysfunction and insulin resistance following lipid infusion. Other clinical trials (NCT00771901; NCT00167934; NCT00298857; https://clinicaltrials.gov/; accessed on 14 January 2022) investigated the effect of sodium phenylbutyrate on insulin sensitivity in adipose tissues, skeletal muscles, and liver, body composition, lipid and glucose metabolism, fatty acid oxidation, endoplasmic reticulum stress indices, and inflammation [147].

Increased histone methylation has been linked to the pathophysiology and consequences of obesity, diabetes, and cardiovascular disease, and there is accumulating evidence that PRMTs play a role in this process [148]. For example, PRMT1 expression and its end product, asymmetrically dimethylated arginine (ADMA), have been linked to diabetic retinopathy and nephropathy in diabetic rats [149]. In addition, silencing PRMT1 restored euglycemic state in the leptin receptor-deficient diabetic mouse model [150]. Therefore, pharmacological inhibition of PRMT1 could alleviate diabetic complications. However, the development of PRMT inhibitors is slow, with only a few available inhibitors such as AMI-1 (7,7′-carbonylbis(azanediyl)bis(4-hydroxynaphthalene-2-sulfonic acid) [151].

Histone demethylation is another epigenetic pathway linked to the development of metabolic disorders. Histone demethylases (HDMs) such as lysine-specific demethylase 1 (LSD1) and LSD2 modulate energy expenditure and gluconeogenic enzymes, mainly G6Pase (glucose 6-phosphatase), FBP1 (fructose-1,6-bisphosphatase), and PEPCK (phosphoenolpyruvate carboxykinase) in adipose tissues [152,153]. Thus, histone demethylase inhibitors are emerging as treatments for obesity, diabetes, and associated comorbidities. Tranylcypromine is a histone demethylase inhibitor with significant adverse effects that have limited its usage to major depressive disorders. In animal models, pharmacological treatment with tranylcypromine inhibited LSDs and promoted the transcription of *FBP1*, *G6Pase*, *C/EBP*, *PPAR*, *PGC1*, and *PDK4* genes involved in gluconeogenesis and energy expenditure [152,153,154].

Several studies have reported the involvement of DNMTs in obesity and associated metabolic and cardiovascular consequences [6]. It has been postulated that methylation of the pancreatic homeobox transcription factor, *PDX1*, regulates pancreatic cell activity. In animal models, inducing DNMT1 activity reduced the expression of the *PDX1* gene, resulting in hyperglycemia and disrupted metabolic function [155]. As a result, it was proposed that the pharmacological suppression of DNMT1 might be considered a viable therapy for obesity and diabetes in which DNMTs are upregulated. However, the use of DNMT inhibitors in treating these disorders is hampered by the significant side effects of the currently available medications. As an alternative, drugs with DNMT inhibitory activity that have been approved by the Food and Drug Administration (FDA) for other indications, such as the antihypertensive, antiarrhythmic medicines hydralazine and procainamide [156,157], are being investigated for their potential impact on treating obesity and diabetes (NCT00000620, NCT02046395; https://clinicaltrials.gov/; accessed on 14 January 2022).

## 4. Conclusions and Future Perspectives

**Figure 1:**
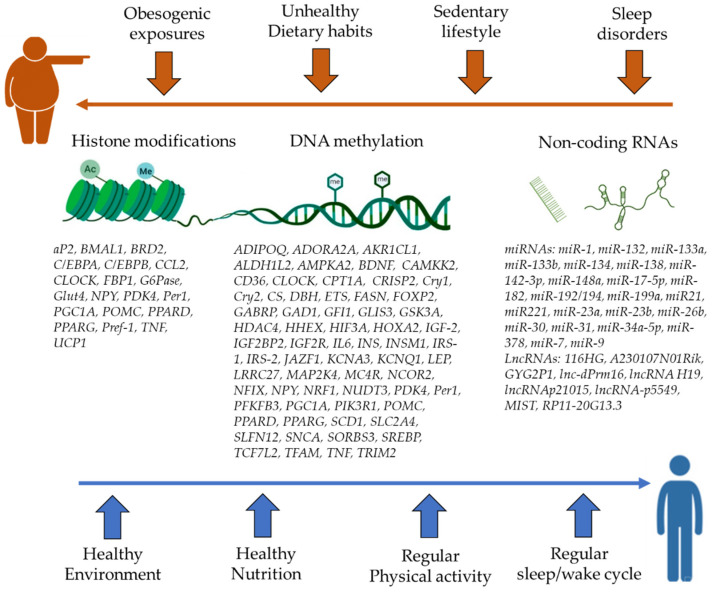
Epigenetic regulations of genes related to obesity. An illustration of the most prevalent epigenetic modifications and targeted genes researched in the context of obesogenic versus healthy lifestyle choices. *ADIPOQ* (adiponectin), *ADORA2A* (adenosine A2a receptor), *AKR1CL1* (aldo-keto reductase family 1 member C8), *ALDH1L2* (aldehyde dehydrogenase 1 family member L2), *AMPKA2* (AMP-activated, alpha 2 catalytic subunit), *aP2* (adipocyte protein 2), BDNF (brain-derived neurotrophic factor), *BMAL1* (brain and muscle aryl hydrocarbon receptor nuclear translocator–like 1), *BRD2* (bromodomain containing 2), *C*/*EBPA* (CCAAT enhancer binding protein alpha), *C*/*EBPB* (CCAAT enhancer binding protein beta), *CAMKK2* (calcium/calmodulin-dependent protein kinase kinase 2), *CCL2* (C-C motif chemokine ligand 2), *CLOCK* (circadian locomotor output cycles kaput), *CPT1A* (carnitine palmitoyl transferase 1A), *CRISP2* (cysteine rich secretory protein 2), *Cry1* (cryptochrome circadian regulator 1), *Cry2* (cryptochrome circadian regulator 2), *CS* (citrate synthase), *DBH* (dopamine beta-hydroxylase), *ETS* (E-twenty-six transcription factor), *FASN* (fatty acid synthase), *FBP1* (fructose-bisphosphatase 1), *FOXP2* (forkhead box P2), *G6Pase* (glucose-6-phosphatase), *GABRP* (gamma-aminobutyric acid type A receptor subunit pi), *GAD1* (glutamate decarboxylase 1), *GFI1* (growth factor independent 1 transcriptional repressor), *GLIS3* (GLIS family zinc finger 3), *GLUT4* (glucose transporter 4), *GSK3A* (glycogen synthase kinase 3 alpha), *GYG2P1* (glycogenin 2 pseudogene 1), *HDAC4* (histone deacetylase 4), *HHEX* (hematopoietically expressed homeobox), *HIF3A* (hypoxia-inducible factor 3 subunit alpha), *HOXA2* (homeobox A2), *IGF-2* (insulin-like growth factor 2), *IGF2BP2* (insulin-like growth factor 2 mRNA-binding protein 2), *IGF2R* (insulin-like growth factor 2 receptor), *IL6* (interleukin 6), *INS* (insulin), *INSM1* (insulinoma-associated 1), *IRS-1* (insulin receptor 1), *IRS-2* (insulin receptor 2), *JAZF1* (Juxtaposed with another zinc finger protein 1), *KCNA3* (potassium voltage-gated channel subfamily A member 3), *KCNQ1* (potassium voltage-gated channel subfamily Q member 1), *LEP* (leptin), *LRRC27* (leucine-rich repeat containing 27), *MAP2K4* (mitogen-activated protein kinase kinase 4), *MC4R* (melanocortin 4 receptor), *MIST* (Macrophage Inflammation-Suppressing Transcript), *NCOR2* (nuclear receptor corepressor 2), *NFIX* (nuclear factor I X), NPY (neuropeptide Y), *NRF1* (nuclear respiratory factor 1), *NUDT3* (nudix hydrolase 3), *PDK4* (pyruvate dehydrogenase kinase 4), *Per1* (period circadian regulator 1), *PFKFB3* (6-phosphofructo-2-kinase/fructose-2,6-biphosphatase 3), *PGC1A* (PPARG coactivator 1 alpha), *PIK3R1* (phosphoinositide-3-kinase regulatory subunit 1), *POMC* (proopiomelanocortin), *PPARD* (peroxisome proliferator-activated receptor delta), *PPARG* (peroxisome proliferator-activated receptor gamma), *Pref-1* (DLK1; delta like non-canonical Notch ligand 1), *SCD1* (stearoyl-coenzyme A desaturase 1), *SLC2A4* (solute carrier family 2 member 4), *SLFN12* (schlafen family member 12), *SNCA* (synuclein alpha), *SORBS3* (sorbin and SH3 domain containing 3), *SREBP* (Sterol regulatory element binding protein), *TCF7L2* (transcription factor 7 like 2), *TFAM* (transcription factor A, mitochondrial), *TNF* (tumor necrosis factor), *TRIM2* (tripartite motif containing 2), *UCP1* (uncoupling protein 1).

Epigenetics is a fast-expanding field of study, and the initial steps toward discovering possible biomarkers for obesity are already being taken. Overall, substantial progress has been achieved in understanding the role of epigenetics in translating the surrounding obesogenic environment to distinct functions and phenotypes (Figure 1). Yet, obesity is a complex, multifactorial disease characterized by an intricate interplay of numerous pathways linked to inflammation, metabolism, oxidative stress, hypoxia, and others. Therefore, there is still much to learn before we entirely comprehend the epigenome’s role in obesity. To elucidate the epigenetic mechanisms that influence adipogenesis, glucose and lipid metabolism, insulin production, signaling, and appetite regulation, well-designed and controlled mechanistic and in vivo studies are required. These studies must explore the epigenetic consequences of hypoxia, inflammatory cytokines, reactive oxygen species, dyslipidemia, elevated glucose levels, hormonal imbalance, and endocrine disruptors. Furthermore, large, prospective research is required to determine whether changes in obesity risk variables, such as food, physical activity, sleeping patterns, alcohol consumption, and other environmental factors, are linked to changes in DNA methylation, chromatin modifications, or microRNA profiles. The modifiable nature of the epigenome should put it at the top of the list of therapeutic targets for obesity and its related comorbidities. Eventually, this may aid in anticipating an individual’s obesity risk at an early stage, opening the door to implementing customized obesity prevention treatments.

## Data Availability

Not applicable.
